# Hash

**Published:** 1875-05

**Authors:** 


### HASH.

(Communicated.)

Oh, Mr. Hall, or whoever you are, how could you say such dreadful things about hash! To be told (as we were in your February number) that our favorite breakfast dish was founded, as it were, upon our old boots and shoes, chopped and made *hard* by stewing, and *therefore*, by some occult means, becoming *soft*, and consequently "as abominable a load for the average stomach as can well be devised." But I think you labor under some delusion. Perhaps you have not been fortunate in your boarding-house or your cook. *We* do not make our hash out of leather. We slice our cold roast beef, and chop it. Sometimes we boil a nice, lean piece of beef till it is absolutely tender, and have it for dinner; and the next morning we hash what is left, carefully excluding all hard, gristly, or knubby scraps, if any such there be.

With us, I have never observed, in any stage of its preparation the phenomenon you mention under the phrase "hardens the surface of each particle;" but then I never have used a microscope in my culinary investigations; a common fork has been my crucial implement.

If all you say is true, what do you mean by recommending hash as "reasonably good food for cats and dogs?" We belong to a genteel family, and *never* bolt our food. Our dog, however, has no inherited fastidious scruples, and *does* bolt his. Now if the unwholesomeness of hash consists chiefly in the "tendency to allow it to slip down the throat unchewed," how can I consider that I put it to its "best use" if I "pass it gracefully over" to poor Carlo?

We have always supposed our hash to be a very wholesome article of food ; we certainly eat it frequently, and seem to thrive upon it ; but if you can convince us, by any plain argument, that we are doing ourselves an injury, I shall thenceforth banish it from my cuisine, and not even prepare any for my "cats and dogs" till I have interviewed Mr. Bergh and obtained his opinion. FELICITE L. D.

[It is very evident that the delicious and savory compound described by our fair friend, is not the "average hash" of which we spoke in February. *That* was, and is, and ever shall be, fit only for cats and dogs, whose gastric juice dissolves ordinary leather with great facility. "Average hash" is tough meat, chopped just fine enough to slip down the throat without chewing, and with the surface of each particular particle still further toughened by the nice little tanning-process to which it is subjected in the course of preparation. Such as this is not fit food for refined people. While we commend hash prepared after the first recipe as palatable and not without nourishment, we adhere to our oft-repeated assertion, that food prepared for swallowing without the necessity of chewing, is bad for the stomach and bad for the teeth and gums.

Digestion in the human animal begins in the mouth—the alkaline saliva dissolving portions of food for which the acid gastric-juice is an inadequate solvent. If food is so comminuted and lubricated in the process of cooking, as to allow it to slip into the stomach unmasticated, it is not wholesome food. It may, however, be very nourishing for some species of animals who have no molar teeth with which to grind their food, and with whom digestion begins and ends with the action of gastric-juice of enormous power.]

TYPHUS AND TYPHOID.—One of the most remarkable points of contrast between typhoid and typhus fevers is to be found in the different classes of society attacked by these fevers. The frequency with which the former prevails among the higher classes of society is being constantly brought home to us, and it would almost seem as if the habits of life and the varied, rich, and plentiful diet of the more opulent classes induced a condition of susceptibility to the disease ; indeed, it has been contended, with some show of plausibility, that just as the liability to an attack of typhoid fever at certain ages corresponds with the condition of greatest physiological activity in the intestinal glands, so the increased functional activity of the same structures, under the stimulus of what is styled "good living," tends to increase the susceptibility of the system to the fever poison. Some authorities have even gone so far as to assume that there is in many persons an inherent predisposition to typhoid fever, allied to that recognized in some other constitutional maladies. However this may be, it is certain that while typhus commits its ravages among the poor, the destitute and the overcrowded portions of the community, the case is far otherwise with typhoid fever, which, while it is no respecter of persons, affecting all classes and occurring in almost all climates, prevails to a remarkable extent among the upper and well-to-do sections of society. We think that the subject of the influence of station of life, with all that this implies in regard to differences of diet and mode of living, is one worthy of attention.

---

Drink ale or lager moderately, and you will soon be a lovely dyspeptic.

